# Financial Health of Private Equity-Backed Groups: Perspectives From Eye Care

**DOI:** 10.7759/cureus.39582

**Published:** 2023-05-27

**Authors:** Sarishka Desai, Rohail Memon, Evan Chen, Sachi Patil, Daniel Vail, Sailesh Konda, Ravi Parikh

**Affiliations:** 1 Department of Ophthalmology, University of Connecticut School of Medicine, Farmington, USA; 2 Department of Dermatology, Northwestern University Feinberg School of Medicine, Chicago, USA; 3 Ophthalmology, University of California San Francisco, San Francisco, USA; 4 Department of Ophthalmology, New York University Langone Health, New York, USA; 5 Department of Ophthalmology, New York Eye and Ear Infirmary of Mount Sinai, New York, USA; 6 Department of Dermatology, University of Florida College of Medicine, Gainesville, USA

**Keywords:** corporatization, debt, optometry, ophthalmology, private equity

## Abstract

Background: In private equity (PE) buyouts of medical practices, it is common for the PE firm to raise significant levels of debt in order to finance the purchase. This debt is subsequently shouldered by the acquired practice(s). There remains a scarcity of literature quantifying the effect of PE acquisition on the subsequent financial performance of eye care practices. We aim to identify and characterize debt valuations of ophthalmology and optometry private equity-backed group (OPEG) practices, which serve as an indicator of practice financial performance.

Methods: A cross-sectional study from March 2017 to March 2022 was conducted using business development company (BDC) quarterly/annual filings to the Securities and Exchange Commission (SEC). The 2021 BDC Report was used to identify all BDCs actively filing annual reports (Form 10-Ks) and quarterly reports (Form 10-Qs) in the United States in 2021. The public filings of BDCs lending to OPEGs were searched from the inception of the OPEG’s debt instrument in a BDC’s portfolio and the amortized cost and fair value of each debt instrument were tabulated. A panel linear regression was used to evaluate temporal changes in OPEG valuations.

Results: A total of 2,997 practice locations affiliated with 14 unique OPEGs and 17 BDCs were identified over the study period. Debt valuations of OPEGs decreased by 0.46% per quarter over the study period (95% CI: -0.88 to -0.03, P = 0.036). In the COVID-19 pre-vaccine period (March 2020 to December 2020), there was an excess (additional) 4.93% decrease in debt valuations (95% CI: -8.63 to -1.24, P = 0.010) when compared to pre-pandemic debt valuations (March 2017 to December 2019). Effects of COVID-19 on valuations stabilized during the pandemic post-vaccine period (February 2021 to March 2022), with no change in excess debt valuation compared to pre-pandemic baseline (0.60, 95% CI: -4.59 to 5.78, P = 0.822). There was an increase in practices that reported average discounted debt valuations from 20 practices (1.6%) associated with one OPEG to 1,213 practices (40.5%) associated with nine OPEGs (including 100% of newly acquired practices), despite the stabilization of COVID-19-related excess (additional) debt.

Conclusions: Debt valuations of eye care practices have declined significantly post-PE investment from March 2017 to March 2022, suggesting that the financial health of these groups is volatile and vulnerable to economic contractions such as the COVID-19 pandemic. Eye care practice owners must consider long-term financial risks and impacts of subsequent patient care when selling their practice to a private equity group. Future research should assess the impact of secondary transactions of OPEGs on the financial health of practices, practitioner lifestyle, and patient outcomes.

## Introduction

Private equity (PE) firms are asset managers that raise capital to obtain significant ownership in private companies. PE firms seek to improve the valuations of companies in their portfolios with the ultimate goal of selling these companies at a profit and hopefully generating above-market returns. Investments often have a short life span, from three to seven years from acquisition to turnover [1]. PE acquisition continues to play a greater role in healthcare in the United States, particularly affecting procedure-based, outpatient-heavy fields such as dermatology, urology, and ophthalmology [2-4].

PE investment has implications not only for practice management strategy but also for the long-term economic well-being of practices. PE buyouts often involve raising substantial amounts of debt, often 70% to 80% of the purchase price, to meet the cost of acquisition. Debt financing allows PE firms to commit very little of their own capital (~2% of purchase price) and achieve high internal rates of return (~20%) in order to resell the practice at a profit [1]. However, this debt is carried by the practice, which shoulders the burden of repayment post-acquisition. The financial strength of practice, along with market forces and interest rates, dictates its debt valuation [5]. Interest rates have returned to pre-pandemic levels as of June 2021, minimizing that the possibility external market factors are contributing to the valuation of debt [6]. Thus, the debt valuation of an ophthalmology/optometry PE-backed group (OPEG) can be used as a proxy for overall financial performance [7,8].

Unfortunately, there is minimal reliable information about PE funds, the deals in which they engage, and the debt they hold. Business development companies (BDCs), funds that invest in the debt of developing companies, are an exception [9]. Unlike traditional PE funds which are limited to a select group of private investors, BDCs are traded on public stock exchanges and accessible to non-professional investors [9]. Similar to publicly traded companies, a BDC must file quarterly and annual reports with the Securities and Exchange Commission (SEC). Significant parameters related to debt, including the amortized cost of debt (net debt accumulated) and the fair value (estimated market value), are characterized in these reports [10]. Studying debt reported by BDCs, as previously reported in dermatology literature, can give us insight into trends of business acquisitions and correlated financial performance of OPEGs [7]. The purpose of this study is to investigate how the debt valuations of OPEGs have fluctuated, if at all, since 2012, when our group previously reported a rapid increase in PE-backed acquisitions of ophthalmology and optometry practices [11]. Further, we sought to investigate how these debt valuations were impacted during the COVID-19 pandemic and COVID post-vaccine period, which demonstrated the normalization of ophthalmologic procedural volume to pre-pandemic levels [12]. This article was previously posted to the medRxiv preprint server on November 17, 2022.

## Materials and methods

Data collection

The BDC Report for 2021 was searched for publicly available BDCs, which were reviewed to find BDCs actively filing Form 10-Ks (annual business disclosure reports) and 10-Qs (quarterly reports) [10]. Seventy-nine BDCs with active quarterly and annual filings were identified, and their respective 10-Ks and 10-Qs were searched for the terms “ophthalmology,” “optometry,” “healthcare”, “eye, and “vision” to identify debt held by ophthalmology/optometry groups. Manual searches of news sites, OPEG websites, and the financial database Pitchbook were conducted to identify eye care practices backed by PE firms, and these groups were selected for inclusion in further analysis. Of note, Site for Sore Eyes and Sterling Optical are large, consolidated groups excluded from analysis as they are subsidiaries of Emerging Vision and not PE-owned.

The amortized cost and fair value of each OPEG’s debt instrument in a BDC’s portfolio were tabulated from the inception of the OPEG’s debt instrument in a BDC’s portfolio. A number of practice locations associated with each OPEG were identified by searching each platform’s website. To ensure consistency, the locations of each OPEG as of July 2022 were reported.

Valuation of OPEG debt

The valuation of the total debt of an OPEG (premium or discount) at a given time was calculated by dividing the difference between the fair value and amortized cost by the amortized cost. Factors influencing the fair value (estimated market value) of debt include interest rates, company performance/cash flow, value of company assets, and debt covenants [7]. A premium on the debt, meaning that the fair value of the debt exceeds the amortized debt, means the debt is high quality and will continue to generate income until it matures [13]. Debt valued at a discount, where the fair value is lower than the amortized cost, is a sign that the debt is of lower quality and that an OPEG is perceived as being at higher risk of defaulting on its debt [14].

A panel linear regression was conducted to test for differences in debt valuations across fiscal quarters; individual OPEG debt obligations were used as the unit for the panel. Dummy variables representing the pre-pandemic period (March 2017 to December 2020), pandemic pre-vaccine period (February 2020 to December 2020), and pandemic post-vaccine period (February 2021 to March 2022) were included in the model to account for the specific impacts of each economic environment on debt valuations (excess debt due COVID-19 during those time periods). P < 0.05 was used as the cutoff for significance for all statistical tests. Statistical analysis was performed in February 2023 using Microsoft Excel 2016 (Microsoft Corporation, Redmond, Washington, United States) and R, version 4.0.5 (R Group for Statistical Computing, Indianapolis, Indiana, United States).

## Results

**Figure 1:**
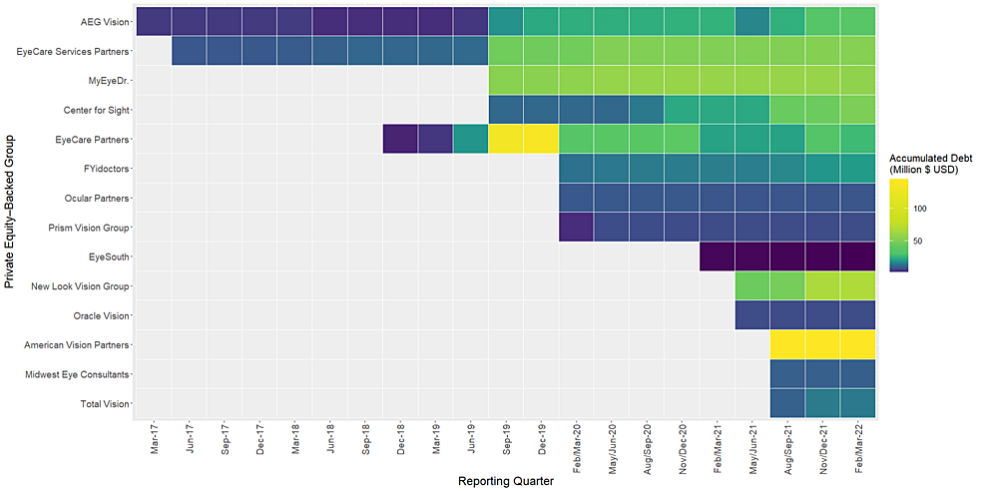
Total Amortized Cost of PE-Backed Ophthalmology and Optometry Debt Instruments Graph depicting the total debt held by each ophthalmology and/or optometry PE-backed eye care group (OPEG), by reporting quarter from March 2017 to February/March 2022. Some quarters are reported as having a range of months as BDCs may file a report (10-K or 10-Q) with the SEC on different dates within the same economic quarter. PE: private equity.

A total of 2,997 practice locations represented by 17 BDCs and affiliated with 14 unique OPEGs were identified in 2021, with associated debt data available from March 2017 through March 2022. As more than one BDC can hold debt in the same OPEG, a total of 32 unique debt obligations were analyzed in this study. In February/March of 2022, the last quarter of data publicly reported during data analysis, a total of 492.9 million USD of amortized debt was held by the study sample. Total amortized cost of loans associated with an individual OPEG during the study period ranged from a low of 1.6 million USD (EyeSouth, February/March 2022) to 144.2 million USD (American Vision Partners, August/September 2021) (Figure 1).

Of all reported debt in 2021, eight debt instruments were acquired during the pandemic pre-vaccine period (March 2020 to December 2020) and 12 additional debt instruments were acquired during the post-vaccine period (February 2021 to March 2022). These acquisitions represent a 167% increase from the 12 OPEG debt instruments reported pre-pandemic, representing rising PE interest in ophthalmology and optometry practices. A plurality of BDCs (47%) held only one debt instrument in their portfolio with 35% holding two instruments, and 18% holding three or more OPEGs in their portfolio during the study period. OPEGs were associated with over 2,997 practice locations throughout the United States and Canada as of July 2022 (Table 1).

**Table 1:** Number of OPEG Practice Locations (July 2022) OPEG: ophthalmology and/or optometry private equity-backed group.

OPEG	Locations
AEG Vision	296
Eyecare Services Partners	78
MyEyeDr.	839
Center for Sight	20
EyeCare Partners	650+
FYidoctors	266
Ocular Partners	9
Prism Vision Group	80
EyeSouth	132
New Look Vision Group	466
Oracle Vision	14
American Vision Partners	60+
Midwest Eye Consultants	38
Total Vision	49

**Figure 2:**
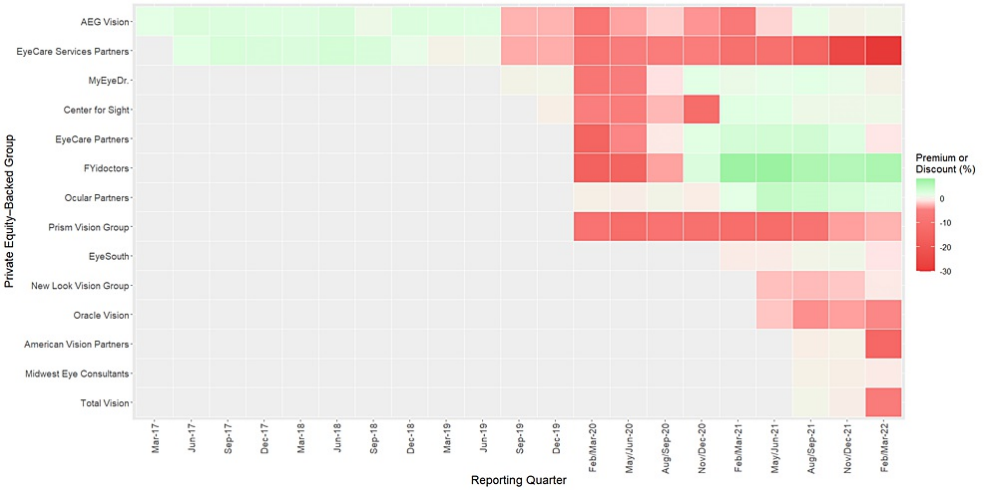
Premium/Discount of PE-Backed Ophthalmology and Optometry Debt Instruments Graph depicting the premium or discount on the debt held by each ophthalmology and/or optometry PE-backed eye care group (OPEG), by reporting quarter from March 2017 to February/March 2022. Some quarters are reported as having a range of months as BDCs may file a report (10-K or 10-Q) with the SEC on different dates within the same economic quarter. PE: private equity.

The valuation of debt instruments was found to be highly variable for many OPEGs during the study period (Figure 2). Panel linear regression analysis revealed the overall valuation of OPEG debt decreased by 0.46% per quarter (95% CI: -0.88 to -0.03, P = 0.036) (Table 2). Despite this downward trend, OPEGs successfully maintained premium or close-to-par debt valuations from March 2017 to December 2022 (pre-pandemic period), meaning that practices were performing well financially. Acuity Eyecare, MyEyeDr., Eyecare Services Partners, and CFS Management all reported average pre-pandemic debt valuations between -0.1% and 1.3%.

**Table 2:** Panel Regression Model on OPEG Debt Valuation CI: confidence interval, p-value: probability value, OPEG: ophthalmology and/or optometry private equity-backed group.

Predictor	Change in Valuation (%)	95% CI (%)	p-value
Time (Economic quarter)	-0.46	-0.89 to -0.03	0.036
Pandemic pre-vaccine (March 2020 to December 2020)	-4.93	-8.63 to -1.24	0.010
Pandemic post-vaccine (February/March 2021 to February/March 2022)	0.60	-4.59 to 5.78	0.822

In addition to this baseline decline in debt valuations, the economic environment of the COVID-19 pre-vaccine period contributed to an excess (additional) 4.93% decrease in valuations (95% CI: -8.63 to -1.24, P = 0.010) when compared to pre-pandemic. During this pandemic pre-vaccine period, 100% of OPEGs reported an average discounted debt valuation (Table 2). Three OPEGs, EyeCare Partners, MyEyeDr., and FYidoctors, were able to return to premium debt valuations by November/December 2020. This economic shock on debt trends stabilized by the post-vaccine period (February 2021 to March 2022), with no significant change in excess valuations when compared to pre-pandemic levels (0.60, 95% CI: -4.59 to 5.78, P = 0.822). Despite the stabilization of excess debt valuations, the baseline decline in valuations persisted over time. During the post-vaccine period, 1,213 practices (40.5% of all practices) associated with nine OPEGs reported an average discounted debt valuation. This represents a 25-fold increase in discounted practices when compared to the pre-pandemic period.

Acquisition of debt instruments increased over time, increasing debt assumed by eye care practices as a whole. Debt acquisition was primarily associated with larger OPEGs with practice locations in multiple states. Compounding the issue of increasing debt, 759 practices associated with six OPEGs acquired in the post-vaccine period reported average discounted debt valuations, signaling poor financial performance of these groups following PE investment. Additionally, the debt associated with EyeCare Services Partners has plunged into the deep discount classification for the first time (> 20% discount) in the post-vaccine period, indicating an extremely high risk of defaulting on debt and potential for bankruptcy [15].

## Discussion

To our knowledge, our study is the first to assess the debt valuations of PE-backed eye care practices. Debt associated with the majority of individual OPEGs in 2021 has declined over time, despite the normalization of economic impacts on debt from the pre-pandemic to the pandemic post-vaccine period. Similar findings were appreciated in a study of debt valuations of PE-backed dermatology groups [7]. Discounted valuations of debt raise concerns about the financial viability of many PE-backed practices, as these valuations indicate practices are less valuable.

Given the significant costs of capital-intensive equipment (e.g., optical coherence tomography machines, ultra-wide fundus photography cameras, slit lamps) in combination with declining reimbursements, accepting PE investment is sometimes viewed as a low-risk financial decision for physician practice owners. However, PE firms invest little of their own capital and are not vulnerable to major losses if an OPEG declares bankruptcy. In contrast, the OPEG itself is directly responsible for servicing the debt incurred by the PE firm. Holding large debt loads, particularly during periods of economic contraction, can place additional financial stress on OPEGs to service interest payments and can put practices at risk for cost-cutting, restructuring, or even bankruptcy [16]. The inherently distorted incentive structure in debt acquisition and payoff between practices and a PE firm often creates conflicts in practice management strategy. Factors that have been cited include loss of autonomy, decreased influence on practice culture, increased scope of midlevel practitioners, reduced yearly compensation, and elimination of the right to due process during the termination of employment [17,18].

A failure to repay debt results in either restructuring the terms of the debt agreement or, in the most extreme situations, bankruptcy of the practice. Physicians usually receive a combination of an initial cash payout and equity when selling their practices to PE firms. In restructuring or bankruptcy, debt investors have a preference over equity holders in claims on a company’s assets [19]. Thus, physicians typically have the last claim on assets if a company liquidates or recapitalizes to pay off debt and consequently assume a higher level of risk than debt investors when their practice is acquired. Unfortunately, early-career physicians shoulder the highest level of financial risk. After a practice is acquired by an OPEG, they typically do not receive the initial cash payoff that practice partners receive and they work at a reduced income potential [20]. An increasing number of early career physicians are entering the field as autonomy and ownership continue to diminish, which may further impact physician leverage in future PE negotiations.

Skeptics of the PE model further point to examples in which short-term cash flows have been prioritized at a detriment to not only physician autonomy but also patient outcomes. Physicians are provided financial incentives by the PE firm that may lead to over- or under-treatment of patients; PE-backed dentistry groups were found to bill for an increased volume of procedures regardless of medical necessity [21]. PE-backed nursing homes exhibit increased short-term mortality rates which may be related to lowered nursing-staff-to-resident ratios and the diversion of patient care funding, both strategies used by PE firms to increase revenue margins [22]. PE investment in healthcare has even entered the public arena, with articles in the New Yorker as well as in Bloomberg Businessweek based on concerns surrounding patient outcomes and medical decision making in addition to potential anti-trust issues [23,24].

Initial findings in ophthalmology have been inconclusive, with some articles indicating minimal changes to patient care and others indicating a rising movement toward short-term profits, such as prescribing more expensive medications [25]. Recently, a difference-in-differences study of PE-acquired dermatology, gastroenterology, and ophthalmology physician practices and independent practices found the former was associated with differential increases in allowed amount and charges per claim, volume of encounters, and new patients were seen, as well as some changes in billing and coding [26]. Further data is required to evaluate PE-backed investments in ophthalmology and optometry, such that quality of care does not decline.

Despite falling valuations and skepticism, PE involvement in eye care is growing at an exponential rate. Investment activity began in the early 2010s, and the market share of PE in optometry and ophthalmology now stands at 16.5% and 14.5%, respectively [27]. Debt instruments held in BDC portfolios from December 2020 to March 2022 represent a 167% increase from the 12 OPEG debt instruments reported pre-pandemic. The majority of the private equity deals occurring in ophthalmology and optometry are still primary transactions, a practice or group of practices being sold directly to a PE firm. Secondary transactions, when a PE firm sells all or part of its ownership interest, started in 2018 when EyeCare Services Partners was sold for an undisclosed amount [28]. The next secondary transaction occurred in 2019 when EyeCare Partners (the largest eye care group in the U.S.) was sold for 2.2 billion USD by FFL Partners [29]. During the four-year hold period, FFL Partners produced a 65% annualized return, outperforming the 4.9% compound annual growth rate for outpatient services between 2016 and 2019 over 13.3 times [29,30]. Strategies such as consolidation, used to increase practice valuations for secondary transactions, indicate a short-term focus on practice growth followed by recapitalization in contrast to physicians and patients, who have a longer-term relationship with one another.

Limitations

There are several limitations to this analysis. First, debt held by BDCs only represents a portion of loans used in the acquisition of OPEGs; additional data transparency is required to capture the true magnitude of private debt held by all OPEGs. Second, publicly reported fair value of debt is tabulated by individual BDC firms and reported to the SEC; inaccuracies may exist in these calculations. Third, the debt included in this study only includes companies reporting debt in 2021, and the results may not be able to be extrapolated to debt held and repaid by companies in previous years. Lastly, BDCs holding debt instruments for non-PE-backed optometry and ophthalmology corporations were excluded from the study, potentially underestimating debt devaluations in the specialty at large.

## Conclusions

Private equity in eye care is a relatively new field of investment; consequently, there is currently little evidence surrounding the ramifications of PE ownership on the longer-term financial outlook and patient outcomes of acquired practices. This study’s findings of decreased practice valuations with increasing debt raise significant concerns surrounding the impact of PE on eye care practices, other types of medical practices, physicians, and, most importantly, patients. Eye care practice owners should carefully consider the long-term financial risks involved when selling their practice to a private equity group. Future studies should examine the impact of high inflation and rising interest rates on the debt valuations of OPEGs as private markets tend to lag public markets. Additionally, studies should focus on the relationship between secondary transactions of OPEGs and the financial health of these practices, as well as providing quantitative evidence on the effects of PE investments on physician autonomy, outpatient procedure volumes, and patient outcomes.
